# Evaluation of a Tracking System for Patients and Mixed Intravenous Medication Based on RFID Technology

**DOI:** 10.3390/s16122031

**Published:** 2016-11-30

**Authors:** María Martínez Pérez, Guillermo Vázquez González, Carlos Dafonte

**Affiliations:** 1 Department of Information and Communications Technologies, Faculty of Computer Science, Campus Elviña S/N, University of A Coruña, A Coruña E-15071, Spain; dafonte@udc.es; 2 Complejo Hospitalario Universitario, A Coruña 15006, Spain; Guillermo.Vazquez.Gonzalez@sergas.es

**Keywords:** RFID, evaluation, traceability, safety, adverse events

## Abstract

At present, one of the primary concerns of healthcare professionals is how to increase the safety and quality of the care that patients receive during their stay in hospital. This is particularly important in the administration of expensive and high-risk medicines with which it is fundamental to minimize the possibility of adverse events in the process of prescription-validation-preparation/dosage-dispensation-administration of intravenous mixes. This work is a detailed analysis of the evaluation, carried out by the health personnel involved in the Radiofrequency Identification (RFID) system developed in the Day Hospital and Pharmacy services of the Complejo Hospitalario Universitario A Coruña (CHUAC). The RFID system is evaluated by analyzing surveys completed by said health personnel, since their questions represent the key indicators of the patient care process (safety, cost, adequacy with the clinical practice). This work allows us to conclude, among other things, that the system tracks the patients satisfactorily and that its cost, though high, is justified in the context of the project context (use of dangerous and costly medication).

## 1. Introduction

The safety of the patient [1,2] is one of the factors which reflects the level of quality in health services and is considered a priority area in healthcare. Assuring this safety is becoming increasingly complicated given that there is a range of diverse potential risks, with no single method capable of guaranteeing a totally risk-free environment. It should be pointed out that healthcare tasks involve factors inherent to the environment combined with those factors relating to human behavior and actions [3,4,5,6]. This is a possible cause for the occurrence of what are called adverse events: situations causing harm to the patient during the treatment process which do not originate in the base illness of the patient and can result in significant harm or even death. According to previous studies, 38% of adverse events happen during the prescription-elaboration-validation-dispensation-administration of medication to the patient [5,7,8]. This process, which involves all types of medical personnel, comprises various tasks: the doctor prescribes medications; the pharmacist validates and dispenses them and finally the nursing staff administers them to the patient.

The strategies and risk management involved in the area of patient safety are directed at detecting errors and their systematic recording; the analysis of retrieved information and its transformation into pragmatic knowledge in order to make continued improvements to the services in question. To increase patient safety it is necessary to develop a range of complementary actions in order to:

- prevent adverse events
- identify them
- mitigate the impact of adverse events when they occur

Nowadays, one of the principal concerns in maintaining a high degree of safety in healthcare environments and minimise the occurrence of adverse events, is to obtain the traceability of patients and the binomial patient/medicine prescribed as it is validated, prepared, dispensed and administered. It is also necessary to register waiting times, patient diagnosis and tests, and identify, in real time, the whereabouts of the patient and their medicines (name of the medicine, time of preparation, transport and administration, lot number, expiry date, route, dose and scheduling of medication during the patient’s stay [9]). This process is known as patient traceability and is one of the means that can contribute to guaranteeing patient safety which is equally as important as the obligations of doctors, pharmacists, dentists, nurses and other health professionals. The Spanish Royal Decree 577/2013, 26 July, legislates the regulation of pharmaceutical vigilance with respect to medication for human use.

Thanks to a research project financed by the *Instituto de Salud Carlos III* (Project PI07/90351), in January 2008 the Pharmacy and Computer Science services of the CHUAC started a research line oriented towards the integration of Radiofrequency Identification (RFID) technology in a healthcare environment. The main purpose of said project was to evaluate the application of RFID technology in tracing patients and medication in an Intensive Care Unit [9]. The experience gained allowed us to demonstrate the technical viability of RFID for the mentioned tasks; however, with regard to the economic viability of labelling the medication of the Intensive Care Unit, we concluded that the cost was too high, considering the price of most medications administered there.

A very different case is obtaining the traceability of patients and medication in the case of those drugs which are considered to be of high risk and cost, given that their precise dosage control facilitates the sustainability of the public health service, as is the case with the Day Hospital. We were granted a second research project, also financed by the *Instituto de Salud Carlos III* (Project PI10/02442), focused on medical treatments prepared in the Pharmatechnical Unit of the Pharmacy Service and administered by the Day Hospital at the CHUAC. These treatments are prescribed for rheumatologic, neurologic, and digestive pathologies and developed in line with the pharma-therapeutic profile of the patient. A number of systems have been developed, as studied here, to obtain the traceability of patients and medicines using new technologies [10,11,12] such as RFID and study its effectiveness in routine clinical practice for health personnel in a hospital.

The preparation and dosification of a large part of the medicines administered to patients in the day hospital are carried out by the Pharmatechnical Unit of the Pharmacy Service. The processes are formalised and prior to the implementation of the RFID system under evaluation in this paper, the registration of medicines prepared for each patient (name of medicine, indications, dosage, guidelines, dosing schedule and route) were carried out manually. Likewise, the same registration procedure was carried out for each prepared medicine: the lot number, expiry date of each component, the date of elaboration, lot numbers and the expiry date for the final product. All this was recorded on paper and it was difficult to maintain global traceability of the medicine, especially a long period after preparation. The time of receipt of a prescription, despatch of medication from the pharmacy or its receipt in the day hospital was not registered.

Prior to the development of the RFID system under evaluation, it was impossible to view easily and with surety (as can be done now electronically in a single tag) the key data pertaining to the preparation and administration of intravenous mixtures (Table 1). Another key point in the care process is when the nursing staff administer the medication to the patient: they need to have a practical guide to the conditions for use and administration of the medicines (see table above) and details as to the likely development of the patient with the medication in addition to other information. It is clearly very important for personnel to be able to automatically validate with precision the prescribed medicine and conditions for its administration.

The Day Hospital Service is characterized by a large rotation of patients whose prescribed medicines have pre-established and long-term scheduling (21 days, monthly…). It should be pointed out that all the medicines involved in the system under study in this work, have special characteristics in the process of prescription, preparation and/or administration to the patient. Normally a patient, on arrival at the day hospital, has to comply with various prerequisites prior to the administration of treatment:

- Have had a medicine administration scheduled for that day
- Be in possession of medical tests with positive results
- Have had previously administered any medicine necessary to prepare the patient to receive the prescribed treatment, e.g., paracetamol

When the patient has complied with the prerequisites outlined above, the pharmacy service is requested to prepare the medicine. However, in routine clinical practice, there are specific situations which may compromise the care process. The principal problems detected that can occur are the following:

- While the medication has been received by the day hospital, the patient’s whereabouts in the hospital are unknown
- The medication has gone missing during its transport from the pharmacy to the Day Hospital
- Neither the nursing staff or the patient has been notified of the arrival of the medicine

It should be highlighted that both the Day Hospital and the Pharmacy Service operate within the framework of current quality standards: the FDA “Good Manufacturing Practice”; standards for the correct preparation and quality control of pharmaceutical compounds; and of course at the same level of assurance as other hospitals with a similar environment and level of complexity. In addition, the Pharmacy Service of CHUAC has been ISO 9001:2000 certified since 2005.

Given the characteristics of the medications (the high cost and patient risk factors involved in their administration), the patients and the operation of the Day Hospital, a developed information and alert system was required that would efficiently and securely automate the storage and appointment information and modifications to all the data related to the traceability of patients and medicines. It should be noted that the consumption of medications selected for this study, is estimated to cost CHUAC between four and five million euros for annually. This can be translated as 3190 doses for 511 patients attended to at the Day Hospital annually, and therefore, we believe that the investment required to integrate RFID technology to undertake the abovementioned actions is totally justified.

The information and alert system being evaluated is designed primarily to increase patient safety and the sustainability of the health system through the integration of RFID technology into the prescription-validation-preparation/dosage-dispensation-administration of medications in the CHUAC Day Hospital. It is important to highlight that this system comprises different RFID systems integrated into the Day Hospital and Pharmacy Service [13]. Each one of these has specific functionalities including facilitating traceability of patients and medicines, precise identification of the patient and the prescribed, validated, prepared and administered medicines, verification that the nursing staff are going to administer the correct medicine in the correct way and under the proper conditions of stability, conservation and transport. It is made up of the following subsystems:

1. RFID system to obtain traceability of patients and the RFID transporter: RFID technologies offer a number of solutions for tracking people and the transporter of prepared intravenous compounds. Aeroscout was chosen for the implementation of a Real Time Location System (RTLS) in the Pharmacy Service and the CHUAC Day Hospital, given that it allows the use of the existing WIFI network available in the hospital therefore reducing installation work and associated costs.
2. RFID system for the preparation of medication in the pharmacy service: This system involves the precise identification of intravenous compounds prepared by the pharmacy service which are subsequently administered to patients in the day hospital. Following preparation of the intravenous compound, the nursing staff then label it with a customized 100 mm × 70 mm size passive tag. The tag is then attached to the intravenous compound in a flag position so as to avoid any RFID interference with the liquids. It contains two RFID tags with two different frequencies UHF and Near-field Communication (NFC). The UHF tag (this frequency has been selected because it can be read within the RFID transporter cart) can be located within the RFID system for the traceability of patients unlike the NFC tag (selected to read at short distances) which is designed to function within the RFID system for the administration of medication to the patient. Both systems are described below.
3. RFID system to obtain traceability of intravenous compounds: This system manages the traceability of intravenous compounds during their transport from dispatch in the Pharmacy Service until their delivery to the Day Hospital. When the nursing staff has completed the medication labelling, it is placed in the transporter to be administered to the patient. The trolley is an innovative element as it has antennas in its interior capable of providing in real time, a list of medications with a frequency of retransmission previously defined by the user.
4. RFID system for the administration of medicine to the patient: This system is designed to ensure traceability and increase safety in the administration of medications to the patient in the Day Hospital. It is fundamental that the nursing staff, when administering medication, are able to check that they are working with the prescribed medicine and under the correct conditions for its administration. To do this, a mobile device is used (an Android telephone with an integrated NFC reader) to read the NFC chip of the intravenous compound and then the NHC of the patient. The application then emits alerts to guide the administration of the medicine to the patient: route, scheduling, conditions for administration, expiry date of the compound and indications as to whether or not the medication has been administered or is still prescribed, etc.

The system, in addition, allows healthcare professionals to consult in real time, the whereabouts of medications and patients and therefore inform, in real time, relatives about the situation of the family member and the medicine that is being administered.

## 2. RFID Technology

RFID technology has been implemented in diverse environments providing significant benefits to the development of different processes. The advantages of its integration into the healthcare environment are widely recognized: the FDA is studying its viability for labeling certain types of medicines to avoid counterfeiting.

**Figure 1:**
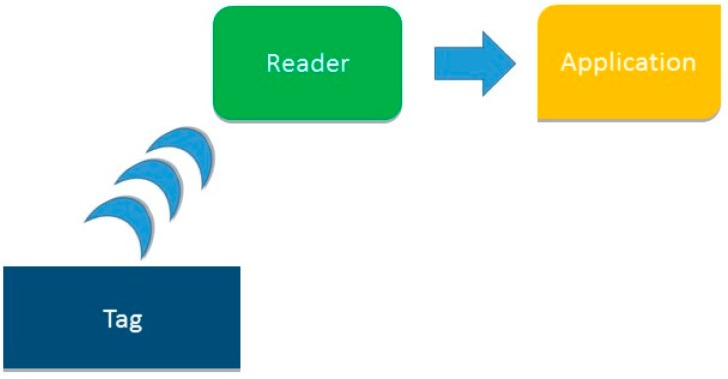
The functioning of RFID technology.

The technology emerged in 1940 and its origin is related to the development of other technologies such as computers, mobile telephones, wireless networks, satellite communications and GPS. The United States military employed an RFID system to remotely identify possible attacks using the classification (Friend or Foe) emitted from any detected plane. At the end of the war, a period of slow but continuous research into this technology began. The publication “Communications by Means of Reflected Power in the Proceedings of the Institute of Radio Engineers” by Harry Stockman in October 1948 can be considered the first research publication into RFID [7]. The diversity of components of RFID technology allows for the building of a large number of different systems. However the working method for all systems is the same: a tag attached to the object or person you want to identify emits a radiofrequency signal with identifying information; a reader receives it and sends the data to a computer application which processes it. This process is illustrated in Figure 1.

The readers can be fixed (antennas, arc-shaped, access points etc.) or mobile (tablets, PDAs, telephones, etc.) The readers can be active if they possess their own energy source for transmitting, passive, if they use the reader as their energy source or semi-active if their own energy source is not used for transmissions but for feeding the internal circuitry of its microchip. There are other significant differences between passive and active tags: cost, available sizes for each of them and the fact that the coverage range is greater with active tags as can be seen in Table 2.

Nowadays, RFID is considered to be a state-of-the-art technology and is deployed in a range of environments. However, a major disadvantage is its elevated cost for certain applications and the lack of automation in some of the processes necessary for its implementation. Therefore, implementing this technology, especially into a healthcare environment with its particular complexity, requires extensive research of those parameters detailed below in order to gain sufficient knowledge to maximize the possibilities and benefits that the technology offers:

- The information to be stored will determine the type of memory in the tag (read-only, write-once or more)
- The tag material: it may deteriorate with use and depends on the aggressiveness of the surroundings
- The shape of tag which depends on the item that is to be identified and/or located
- The adhesive of the tag will depend on the item to be identified and/or located. Special adhesives exist for healthcare products to avoid penetrating, for example, serum or plasma
- The location or protective covering of the tag depends on the item that is to be identified and/or located to avoid any interferences in its readings e.g., attached to materials which contain metal, plastic liquids or glass
- The operating frequency of RFID components which will condition the distance at which the tags can be read

This information facilitates the choice of technical architecture for a specific environment and contributes to the successful implementation of an RFID system. A wide range of identification technologies are now present in the market that could allow for the tracking of patients and medicines in the process of prescription-validation-elaboration-administration of pharmaceuticals by the Pharmacy Service and Day Hospital at CHUAC. However, the success of RFID technology with respect to others principally lies in the extensive capacity of its tags to store information relating to its objects and subjects. This data can also be coded and protected using common standards. Its tags do not deteriorate with use given the variety of materials with which they can be produced such as epoxy which can withstand extreme temperatures (up to −21 °C). Hundreds of tags can be read simultaneously without having to have direct contact with the reader. These properties make RFID an ideal technology for use in tracking systems in healthcare environments (for fridge and room temperature products, to identify patients, unit doses of medicine, diffusion pumps, defibrillators, etc.) The tags are, without doubt, the most important components of the RFIF system. Their design is totally conditioned by the objects or subjects under control. A wide range of tags (size and shape) are available, and together with a suitable protective cover, allow them to be read irrespective of the material to which they are attached.

RFID, as a mature technology, is subject to a series of standards which regulate the interchange of information between devices. These standards, which apply to the identification, reading and exchange of data between devices, are called Electronic Product Code (EPC). EPC global is a consortium of leading organizations in the market and include companies such as Cisco Systems, LG Electronics, Lockheed Martin Corporation and universities, including the Massachusetts Institute of Technology.

The coverage of an RFID component describes the maximum distance at which communication can be maintained with it (to read or write data in its memory, change parameters in the configuration, etc.…). The range depends on other factors besides the operational frequency of the system such as the antenna power of the reader available on the tag, environmental conditions and the direction of the tag when reading, etc. The designated values are, therefore, primarily tentative. An RFID system has four possible operational frequencies as outlined in Table 3.

The operational frequency which functions at 13.56 MHz is now widely used in a range of environments and is now beginning to be deployed in the healthcare environment. This is called NFC and allows for the reading/writing of data in real time on passive RFID tags at a few centimeters distance.

The principal difficulty involved in implementing an RFID system is in the selection of optimum values for the configurable parameters of RFID components. It is, therefore, necessary to evaluate the specific characteristics of each scenario as outlined below:

- Wall materials that may interrupt or reduce radio frequency signals
- Lifts that may complicate the locating of control objects
- Types of rooms (open or semi-open plan)
- The material of the objects to be controlled which may produce interference
- The number and location of access points

RFID will manifest interference with certain materials which complicate its readings, especially if the tags are attached to metals, liquids in plastic or glass. In some cases, it may be necessary to apply a cover to the tags or position them carefully so that readings are error free. The design process of the technical architecture will be largely determined by the experience of the engineers responsible for the project. It is essential therefore to conduct an in-depth study of existing architectures to decide on the most suitable while taking into account the results obtained by other researchers. This involves consultation with the suppliers of RDIF components to acquire different tags and readers to be tested in situ within the system to be developed. Prior to the implementation of the system under study here, more than 150 companies had been contacted and more than 100 articles, published in prestigious international specialist magazines, analysed. This knowledge was essential to be able to develop a suitable and successful design of the architecture which was eventually implemented.

There have been a number of recent developments in the integration of RFID into healthcare environments. Diverse functionalities have been developed which include the implementation of algorithms to augment security in the communication between RFID tags and readers; identification of the patient using RFID for the electronic dispensation of prescribed medicine; mobile applications that facilitate monitoring of patients in their homes; the locating of objects or patients using active and passive tags or active WIFI; automatic RFID dispensers of prescribed medication, etc. These developments all share the same objective to improve safety in the care process of the patient by facilitating the traceability of the patient and the process of prescribing-preparation-dispensing-administering medicine and consequently improving the quality of care received by the patient in the hospital center.

## 3. Objectives and Benefits

The objective of this work was the evaluation of the information and alerts system integrated into the prescription-validation-preparation-dispensation-administration of medication for patients process of the CHUAC Day Hospital [13]. The evaluation was carried out by health professionals from the Rheumatology, Pharmacy, Computer and Day Hospital services at CHUAC, involved in the process of daily clinical practice.

The system was evaluated using different indicators whose values are calculated from the responses to questionnaires given by doctors, specialists in hospital pharmacy, nursing personnel from the Pharmacy Service and Day Hospital, computer engineers from the Computer Service, the subdirector of information systems and patients attended to within this system. The method for calculation of the final value of each of the indicators is detailed below in the methodology section. Through this, the suitability of the system design to daily clinical practice can be analysed together with the advantages and disadvantages detected by the health personnel following their experience of the process. These results will contribute to any actions to be undertaken to improve the system.

## 4. Analysis

The analysis stage focuses on determining which information is most relevant for study to determine the advantages and disadvantages of the system and which professionals will provide more data with respect to each of the indicators following their experience of the system in daily clinical practice. More specifically the analysis can be divided into the following stages:

- The organization of successive meetings with specialists from the Pharmacy, Rheumatology, Computer and Day Hospital services to define those indicators that are most representative for the evaluation of the RFID system in the prescription-validation-preparation/dosage-dispensation-administration process of intravenous mixtures in the Day Hospital. The agreed-upon indicators were as follows:
  ○ Security
  ○ Traceability of the patient
  ○ Traceability of the medication
  ○ Usability
  ○ Efficiency
  ○ Cost
  ○ Suitability for routine clinical practice
- Analyse the capacity of the RFID system to generate information. Determine those modules of the RFID system which provide data to calculate the defined indicators.
- Develop the questionnaires orientated at evaluating the defined indicators for each health professional profile. All sanitary staff implicated in the evaluation of the RFID system is also implicated in the daily practice of the Day Hospital and requires a training that is adapted to the specific features of his/her unit.
- Link the questions in the questionnaire to each of the indicators that they represent.
- Develop the methodology for the calculation of values of the indicators on the basis of the responses to the questions from the professionals, paying particular attention to the functions they perform in the prescription-validation-preparation-dispensation-administration process of medication for patients.

## 5. Design

**Figure 2:**
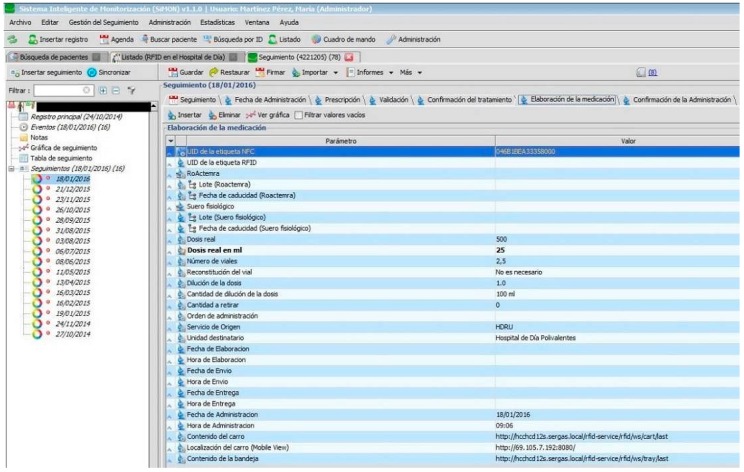
Tracking the preparation of the intravenous mixture.

The design stage has been defined by the route of the patient from entry to hospital until departure. This is necessary so that all RFID systems can provide data relating to the patient’s care attendance in the prescription-validation-preparation/dosage-dispensation-administration process of medication in the Day Hospital. The steps for doctors and patients to follow are as follows:

- If the doctor prescribes medicine for the first time using the RFID in the Day Hospital protocol, it is necessary for the hospital commission in charge to authorize the administration of the medicine to the patient.
- Once authorized, the treatment of the patient can begin with the doctor explaining the procedures with informed consent and once accepted, an Aeroscout active WIFI tag is given to the patient.
- The doctor links, in Mobile View, the patient’s medical history number with the MAC address of the tag which locates it to subsequently obtain traceability of the patient within the system (see Figure 1). This means that for each administration programmed by the doctor, the whereabouts of the patient can be identified in real time: the arrival and exit from hospital and real-time location of the patient in each of the controlled areas: medication treatment room in the Day hospital, waiting room, cafeteria, etc.
- The doctor will, from time to time, program future administrations under the date tag for administrations within the RFID protocol of the Day Hospital (an application which has been developed by the CHUAC Computer Service for the professionals involved) to manage these tasks.
- Prior to the arrival of the patient to hospital, the pharmacist will have prepared the instructions for the preparation of the prescribed medicine for that patient in the tag “Validation” of the RFID protocol of the Day Hospital.
- For each of the programmed administrations, the patient should attend the doctor’s surgery to confirm that his/her state of health is suitable for the treatment to be administered and, if it is, the doctor will notify the pharmacy through the tag “Confirmation of Treatment” under the RFID protocol of the Day Hospital.
- The pharmacy personnel prepares and identifies with precision the intravenous mixture to administer to the patient using the “Preparation of Medication” tag in the RFID protocol of the Day Hospital (see Figure 2) and the RFID system for the preparation of medication of the Pharmacy Service. The medicine will then be placed in the RFID transporter cart and by using the RFID system for tracking medication, the location of the medicine can be identified during its transport from the Pharmacy to the Day Hospital.

When the cart finally arrives at the Day Hospital, the orderly deposits the medication in the RFID tray to control delivery and, then, the nursing staff will administer the medicine to the patient using the “RFID system for the administration of medication”. They identify the patient through the patient medical history number (NHC), read the medication tag and by using the mobile application and its series of alerts, confirm that they are going to administer the prescribed medicine to the correct patient in the correct conditions for administration (dose, route, scheduling, infusion rate, expiry date, etc.).

The number of patients involved has increased on a weekly basis given that the objective is to include all those patients with medicines prescribed by the day hospital and considered to be of high-risk and elevated cost. The most important incidents that have been detected so far relate to problems reading the bar code of the MAC tag (poor orientation of the reader); errors in the placement of the adhesive of the RFID tags on the intravenous mixtures developed by health personnel. These problems have diminished gradually in line with the learning experience gained by the personnel involved. It is important to note that the system is ready to be easily extended to any other prescribed treatment service at CHUAC.

Table 4 illustrates which information is provided for each of the indicators analysed in this study. Seven questionnaires have been designed with one for each professional profile involved in the development and practical use of the system under evaluation. Only simple questions have been presented and only related to tasks with which the professionals are directly involved so that, with their experience, they are likely to respond more accurately.

The responses in 19 questionnaires have been analysed and generated the following results and conclusions.

## 6. Methodology

**Figure 3:**
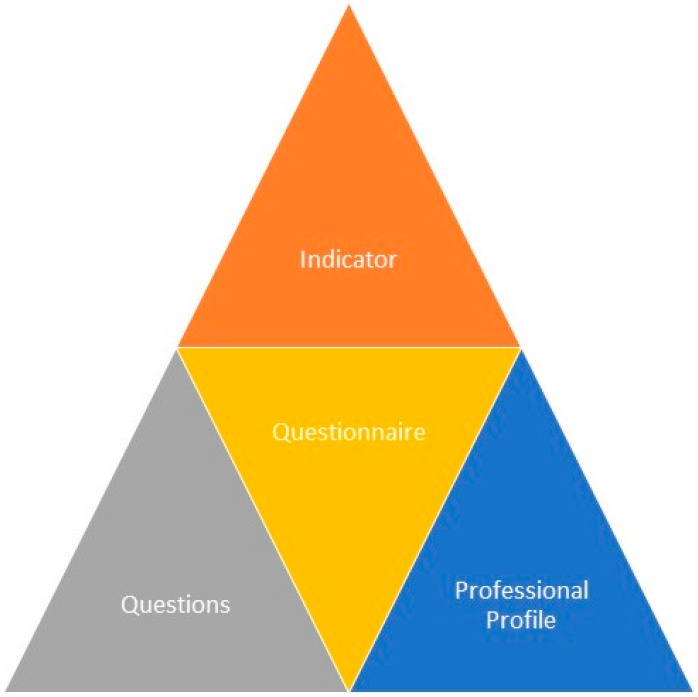
Methodology for calculating the values of the indicators.

The methodology for calculating the values of the indicators (see Figure 3) is based on the responses of professionals and will be weighted according to the functions and levels of responsibility undertaken in the process of prescription-validation-preparation-dispensation-administration of medication for patients.

All questionnaries have been composed of n questions and the professional has to choose a value on a 1-to-5 scale (where 1 reflects total disagreement, 5 reflects total agreement) to indicate the level of agreement with the given statement.

The questions are associated with specific themes and, as such, are only taken into account to find the value of the indicators to which they relate. Finally, the questions are weighted to calculate the value of the indicator, paying attention to the level of responsibility and/or the specialism of the professional involved. The methodology for putting the system into practice has focused on the progressive implementation of each of the RFID systems, to minimise the length of learning time involved for the health professionals concerned and waiting times of the patients involved in this care process.

What is laudable is that nothing was delayed with respect to its normal functioning. In fact, both protocols co-existed for a period of time until the health professionals developed the ability to manage their tasks in minimum time. The number of patients involved in the system has been allowed to increase in proportion to the number of professionals using the new system but only when this did not cause any delays in care times, diagnosis, tests or administration. The methodology allows increasing the number of professionals in the first place followed by the number of patients. Additional support has however been required from the pharmacy service and the supervisor of the day hospital, to assist in synchronizing the tasks of both services.

The surveys were completed in writing or through electronic mail by the sanitary staff (pharmacists, nurses, physicians, supervisors of the Day Hospital) as well as by the Deputy Head of Information Systems. All surveys (19 in total) were completed by members of the staff who are usually involved in the patient care process, during their working hours, and at their usual working place (Table 4 and Table 5). The Day Hospital is a highly specialized unit that administers drugs with very specific preparation and administration conditions, and that are considered dangerous and costly. The nursing and medical staff have therefore ample experience and are specially trained to work in the indicated unit.

## 7. Discussion and Results

**Figure 4:**
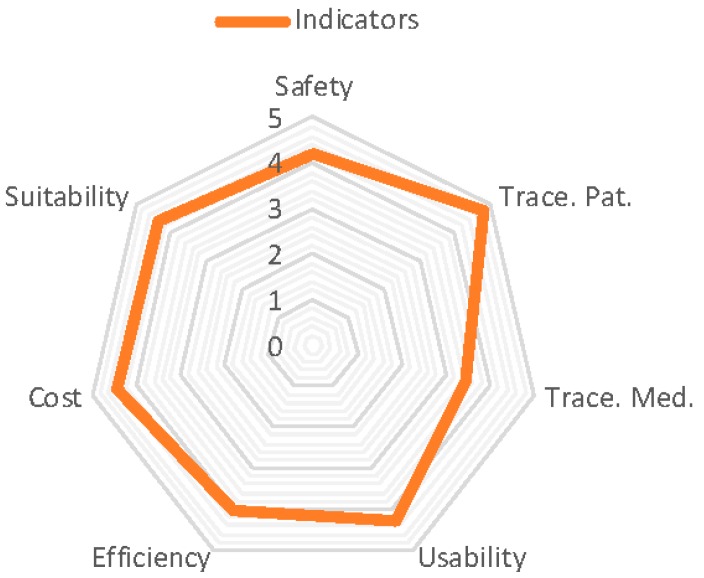
Global values of the analysed indicators with a radial graphic.

The results of the questionnaires have been analysed from two different perspectives. Figure 4 shows the global values for the indicators that are defined in the analysis phase and evaluate the system following the consideration of responses from all the health professionals involved.

**Figure 5:**
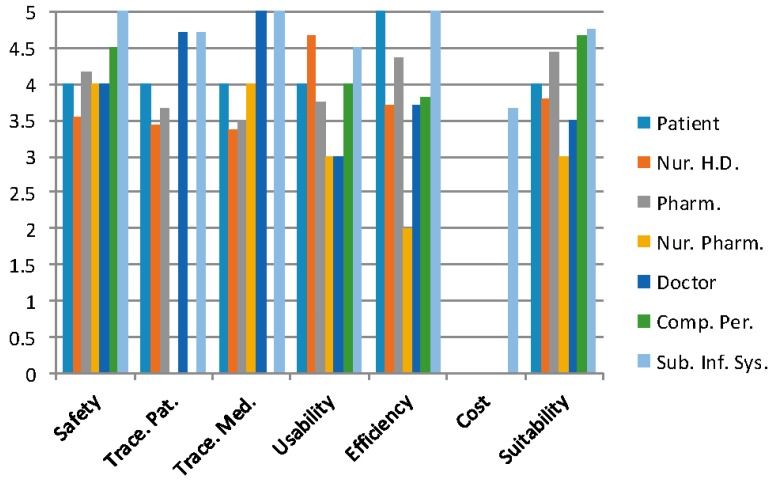
Values of the Indicators analysed for each professional profile.

**Figure 6:**
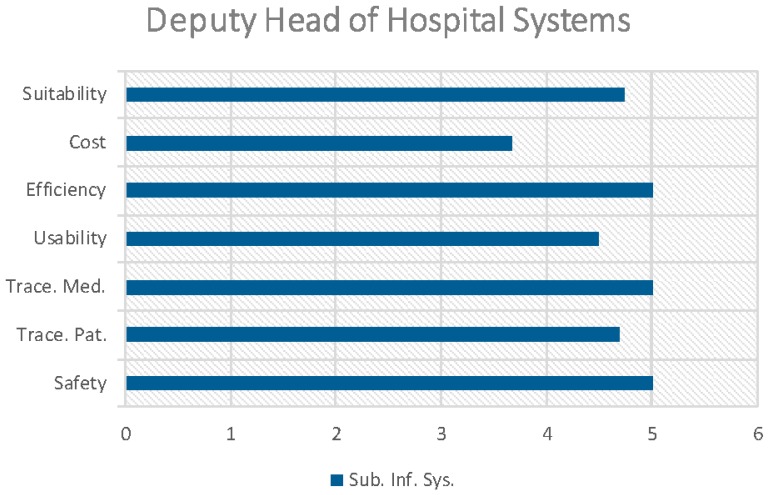
Values of the indicators as analysed for the Deputy Head of hospital systems.

Figure 5 and Figure 6 (only the Deputy Head of hospital systems) illustrate the results of the indicators for each professional profile in the process of prescription-preparation/dosage-validation-elaboration-administration to patients in the CHUAC Day Hospital.

The training period for nursing personnel took less than a month and occupied approximately 2 h daily. It should be noted that the entry and exit of patients to and from hospital might not be identified correctly due to the poor positioning of tags; patients are therefore recommended to wear the RFID tag in a visible position upon entry to the hospital. The costs associated with the loss of tags (there has been one case), especially with elderly patients, should be noted, in addition to the fact that they are not reusable (from one user to another) as they cannot be sterilized in an autoclave. As a result they are given to the patient in a plastic bag with a European cut handle and a neck cord.

Communication between the doctors and pharmacists has improved significantly, given the fact that the prescription and programming of administrations can be carried out immediately. In the same way, the arrival and health condition of a patient can be notified immediately. This allows nursing personnel in the pharmacy to work efficiently and safely in the preparation of medication while minimizing the number of unusable doses, taking into account the brief stability of these medications. Another key point of the system is the ability to control, in real time, the transport of intravenous mixtures with precise identification using computer-assisted surgery situated in the cabin: using NFC and UHF readers integrated as USBs, each medicine can be identified through the dual tag of dual frequency attached by nursing personnel following its preparation.

The lowest value corresponds with the tracking of medication (3.46), which is not surprising given the number of tasks and the precision involved in the preparation process. This includes introducing the lot number and expiry date on each of the components, placing the RFID tag in flag position, placing the mixture in the cart, depositing it in the RFID tray to register delivery time, and finally its administration to the patient.

The nursing personnel experienced an excess of work at the beginning of the learning process, as can be seen in the efficiency indicator (3.71 in their category), but recognized the safety issue, as is shown by the safety indicator in their professional profile (Figure 5). The general learning process of the sanitary staff could be minimized by organizing a series of longer sessions; this would also allow for the use of this system in other hospital services.

The highest value relates to the traceability of the patient (4.78), where the RTLS system is capable of locating to a precision of between 1 and 4 m and less than 5 for the number of patients, especially elderly patients forgetting the device. The second highest value relates to the suitability of the system to routine clinical practice (4.38); we can conclude from this that we must include the collaboration of the majority of future users in the project analysis and the stages of design, implementation, tests, and beginning of the project. Certain adjustments to the system have been made which have caused delays to the project development, but this has contributed to the suitability of the system and its development for routine clinical practice in the hospital. The next section will analyse the results shown in the previous figures and the conclusions drawn.

## 8. Conclusions

We have been able to develop a system of information and alerts using RFID technology integrated into the process of prescription-preparation/dosifcation-validation-elaboration-administration of medication for the patients of the CHUAC Day Hospital, which has notably satisfied the expectations of the involved professionals and patients. For all the defined indicators the value was above 6.9 on a scale of 10.

Importantly, all the qualitative conclusions that are described below correspond to the transcription of observations that were transmitted by the sanitary personnel in writing (in the *Comments* section of the surveys), orally (when handing in the surveys), or even during the development of the present work.

The cost indicator has only been evaluated by the Subdirector of Information Systems who assessed it at 3.67. This is due to the fact that the cost is substantial but it is justified given the context (high cost medication and risk) and the notable increase in both the efficiency of the processes (5) and safety (5) in the care process of the patient (Figure 6).

The system increases patient safety (4.2) by reducing human error and so improves the efficiency of the service. At the same time, it reduces the number of unusable intravenous mixtures (past expiry date or the patient has not arrived for its administration) and so contributes to the sustainability of the health service (the global indicator for medication tracking is 3.46).

There have been significant improvements in the measurement of waiting times, diagnosis and patient tests given that bottle necks can be detected, leading to an overall improvement to the services involved. Safety has improved with the registration of lot numbers, expiry date of components and prepared intravenous mixtures; the additional information provided to nursing staff relating to the administration of medicine (name, route, schedules, dose, indications, etc.), clinical data on the patient and the prepared medicine leading to the clear identification of the binomial patient/prescribed medicine.

Future work will involve the selection of rechargeable RFID devices that can be recharged during the process of administration of intravenous mixtures, and the increase in the number of patients, services, and medication being administered using RFID in the process of prescription-preparation/dosage-validation-dispensing-administration of medication in CHUAC.

## Figures and Tables

**Table 1:** Key parameters of an intravenous mixture.

Key Parameters	
Patient data	Composition
Time of preparation	Pharmacist in charge
Despatch time	Stability
Health professional responsible for preparation	Special conditions for use
Previous medication	Rate of infusion
Lot number and date of expiry of components	Prepared medication

**Table 2:** Comparison between Readers and RFID tags.

Fixed Reader	Mobile Reader	Active Tags	Passive Tags
Antenna	Tablet	Battery	No Battery
Arcs	PDA	More expensive	Less costly with less coverage
Access points	Mobile telephone	Greater size	Longer life

**Table 3:** Operational frequencies in RFID technology.

Operational Frequency	Band
Low Frequency	Below 135 kHz
High Frequency	13.56 MHz
Ultra-High Frequency	433 MHz, 860 MHz and 928 MHz
Microwave	2.45 GHz and 5.8 GHz

**Table 4:** RFID Systems which provide data for the indicators to evaluate.

Indicators	RFID System for the Traceability of Patients and RFID Transporter Cart	RFID System for the Preparation of Medication in the Pharmacy Service	RFID System for Trucking Intravenous Mixtures	RFID System for the Administration of Medication to the Patient
Safety	X	X	X	X
Traceability of patient	X	-	-	X
Traceability of medication	X	X	X	X
Usability	X	X	X	X
Efficiency	X	X	X	X
Cost	X	X	X	X
Suitability for routine clinical practice	X	X	X	X

**Table 5:** Thematic content of the questionnaires developed for each professional profile.

Indicator/Questionnaire	Number of Questions	Safety	Traceability of Patients	Traceability of Medication	Usability	Efficiency	Cost	Suitability for Routine Clinical Practice
Patient	8	X	X	X	X	X	-	X
Doctor	19	X	X	X	X	X	-	X
Pharmicist specialising in hospital pharmacy	36	X	X	X	X	X	-	X
Nursing staff in the day hospital	16	X	X	X	X	X	-	X
Nursing staff in the hospital pharmacy	14	X	-	X	X	X	-	X
Computer personnel	18	X	-	-	X	X	-	X
Subdirector of hospital systems	22	X	X	X	X	X	X	X

## Abbreviations

The following abbreviations are used in this manuscript:

CHUAC	Complejo Hospitalario Universitario A Coruña
RFID	Radio Frequency Identification
UID	Unique Identifier
NFC	Near Field Communication

## References

[1] Osborn S., Williams S. (2004). Seven Steps to Patient Safety.

[2] Kaelber D.C., Bates D.W. (2007). Health information exchange and patient safety. J. Biomed. Inf..

[3] Heinrich H.W. (1941). Industrial Accident Prevention: A Scientific Approach.

[4] De Vries E.N., Ramrattan S.M., Smorenburg Gouma D.J. (2008). The incidence and nature of in-hospital adverse events: A systematic review. Qual. Saf. HealthCare.

[5] Instituto Nacional de Estadística (2008). Establecimientos Sanitarios con Régimen de Internado. http://www.INE.es.

[6] Perrow C. (1986). Complex Organizations: A Critical Essay.

[7] Portillo J., Bermejo A.B., Bernardos A., Casar J.R., Martínez I. (2007). Tecnologías RFID: Aplicaciones en el Ámbito de la Salud.

[8] Gerardo I.C.-R., Mariano C.-C., Lino C., Olaia V., Loli R., Guillermo V. (2010). Co-Morbidity Analysis and Decision Support on Transplanted Patients Using Machine Learning Techniques. Medical and Care Compunetics 6.

[9] Martínez Pérez M., Cabrero-Canosa M., Vizoso Hermida J., Carrajo García L., Llamas Gómez D., Vázquez González G., Martín Herranz I. (2012). Application of RFID Technology in Patient Tracking and Medication Traceability in Emergency Care. J. Med. Syst..

[10] Perrow C.B. (1999). Normal Accidents: Living with High Risk Technologies.

[11] Dantu R., Clothier G., Atri A. (2007). EAP methods for wireless networks. Comput. Stand. Interfaces.

[12] Koshy R. (2005). Navigating the information technology highway: Computer solutions to reduce errors and enhance patient safety. Transfusion.

[13] Pérez M.M., González G.V., Dafonte C. (2016). Safety and Traceability in Patient Healthcare through the Integration of RFID Technology for Intravenous Mixtures in the Prescription-Validation-Elaboration-Dispensation-Administration Circuit to Day Hospital Patients. Sensors.

